# Work Ability Assessment and Its Relationship with Cardiovascular Autonomic Profile in Postural Orthostatic Tachycardia Syndrome

**DOI:** 10.3390/ijerph17217836

**Published:** 2020-10-26

**Authors:** Franca Barbic, Maura Minonzio, Beatrice Cairo, Dana Shiffer, Antonio Roberto Zamuner, Silvia Cavalieri, Franca Dipaola, Nicola Magnavita, Alberto Porta, Raffaello Furlan

**Affiliations:** 1 Internal Medicine, Humanitas Clinical and Research Center—IRCCS, Via Manzoni 56, 20089 Rozzano-Milan, Italy; maura.minonzio@humanitas.it (M.M.); dana.shiffer@humanitas.it (D.S.); franca.dipaola@humanitas.it (F.D.); raffaello.furlan@hunimed.eu (R.F.); 2 Department of Biomedical Sciences, Humanitas University, Via Rita Levi Montalcini 4, 20090 Pieve Emanuele-Milan, Italy; 3 Department of Biomedical Sciences for Health, University of Milan, 20122 Milan, Italy; beatrice.cairo@unimi.it (B.C.); alberto.porta@unimi.it (A.P.); 4 Departamento de Kinesiología, Universidad Católica del Maule, 3480112 Talca, Maule, Chile; beto.zam@gmail.com; 5 Department of Life Sciences & Public Health, Università Cattolica del Sacro Cuore, 00168 Rome, Italy; silvia.cavalieri23@gmail.com (S.C.); nicolamagnavita@gmail.com (N.M.); 6 Department of Woman, Children & Public Health, Fondazione Policlinico A. Gemelli—IRCCS, 00168 Rome, Italy; 7 Department of Cardiothoracic, Vascular Anesthesia and Intensive Care, IRCCS Policlinico San Donato, San Donato Milanese, 20097 Milan, Italy

**Keywords:** autonomic nervous system, heart rate variability, arterial pressure, baroreflex, spectral analysis, head-up tilt, work ability

## Abstract

Postural orthostatic tachycardia syndrome (POTS) negatively impacts quality of life. The excessive increase in cardiac sympathetic modulation during standing, which characterizes POTS patients, leads to many symptoms and signs of orthostatic intolerance. Little is known about the consequences of the disease on work performance and its relationship with individual autonomic profiles. Twenty-two POTS patients regularly engaged in working activity (20 females, age 36 ± 12 years) and 18 gender- and age-matched controls underwent a clinical evaluation and filled out the Work Ability Index (WAI) questionnaire. POTS patients completed the Composite Autonomic Symptom Score (COMPASS31) questionnaire, underwent continuous electrocardiogram, blood pressure and respiratory activity recordings while supine and during a 75° head-up tilt (HUT). A power spectrum analysis provided the index of cardiac sympatho-vagal balance (LF/HF). WAI scores were significantly reduced in POTS patients (29.84 ± 1.40) compared to controls (45.63 ± 0.53, *p* < 0.01). A significant inverse correlation was found between individual WAI and COMPASS31 scores (r = −0.46; *p* = 0.03), HUT increase in heart rate (r = −0.57; *p* = 0.01) and LF/HF (r = −0.55; *p* = 0.01). In POTS patients, the WAI scores were inversely correlated to the intensity of autonomic symptoms and to the excessive cardiac sympathetic activation induced by the gravitational stimulus.

## 1. Introduction

Postural orthostatic tachycardia syndrome (POTS) is an autonomic disorder characterized by a marked increase in heart rate (>30 beats per minute) or tachycardia (>120 beats per minute) upon standing or when exposed to a passive gravitational stimulus (i.e., head-up tilt) without orthostatic hypotension. The disorder usually fasts for more than 6 months. It is more frequent in females (female-male ratio of 5:1) and most cases occur between the ages of 15 and 50 years [1,2,3,4].

Excessive orthostatic tachycardia can be considered a final common pathway that is responsible for most of the orthostatic intolerance symptoms reported by patients [1,2,3,4]. These are related either to cerebral hypoperfusion (lightheadedness, weakness, blurred vision, pre-syncope and, rarely, syncope) [1,4,5] or to excessive sympathetic activation (intolerable palpitations, nausea, tremulousness, and anxiety) [1,2]. Exacerbating factors, some of which are potentially associated with specific working activities, include excessive heat exposure, physical exertion, anxiety, depression, and prolonged bedrest or inactivity [2,3]. The pathophysiological mechanisms of POTS remain poorly understood and three different phenotypes are described: the “hyper-adrenergic” [3], the “neuropathic” [6,7,8] and POTS associated with physical deconditioning [4,9,10] or to a prolonged exposure to gravity absence [4,11,12,13].

The wide spectrum of symptoms experienced by POTS patients are often life-altering and debilitating [14,15,16,17]. People with POTS suffer from increased sleep disturbance and suicidal ideation compared to the general population [18]. They often experience fatigue [19] and cognitive complaints, often referred as “brain fog” [20,21]. In addition, it has been described that this syndrome negatively impacts work performance and patients may be unable to work or study as a direct result of their symptoms [22]. This is particularly relevant for working activities that involve prolonged standing, frequent changes of posture and heat exposure. Finally, the physical deconditioning induced by the orthostatic intolerance sustains and worsens this chronic syndrome by creating a negative vicious circle. The risk of employment loss for these patients is high [22].

As reported by Furlan and colleagues [23], POTS patients are characterized by increased sympathetic outflow to the heart and vessels while supine compared to healthy controls. In addition, during orthostatic stress a marked enhancement of heart rate (HR) and of the spectral index of cardiac sympatho-vagal modulation, LF/HF, compared to controls were observed in the presence of a blunted sympathetic neural discharge to the vessels as quantified by the muscle sympathetic nerve activity. The baroreflex control of HR and of the efferent sympathetic activity to the vessels was preserved. The abnormal cardiac sympathetic response to the gravitational stimulus is strictly correlated with the symptoms of orthostatic intolerance characterizing POTS and to the consequent physical and mental impairment of these patients [1,2].

Until now, little is known about the impact of POTS on working performances. The Finnish Institute of Occupational Health was developed in the early 1980s to assess work ability—the “Work Ability Index” (WAI) [24]. Since then, the WAI has been implemented and widely disseminated in occupational health services [25] and applied in several research activities [26,27].

In the present study, we test the hypothesis that both the severity of the disease as well as the response of the cardiovascular autonomic control of POTS patients to the orthostatic stimulus may be related with their actual working capability as assessed by the WAI.

The rationale of the present investigation is to furnish occupational physicians with useful information for promoting more focused clinical treatments of POTS patients, including physical reconditioning to facilitate adherence to specific work tasks and to set specific and more effective interventions in the workplace.

Based on the above considerations, the aims of the present study were:

1. To evaluate the work ability of POTS patients regularly engaged in working activity compared to a group of healthy controls.
2. To evaluate the roles of the autonomic impairment of POTS patients and their individual cardiovascular autonomic responses to the orthostatic stimulus in work ability.

## 2. Methods

The study protocol adhered to the principles of the Declaration of Helsinki and was approved by the Local Ethical Review Board (Approval # 1611/2016). The study was funded by the Italian Ministry of Health (Grant RF-2013-02355242). All of the study participants provided their written informed consent.

### 2.1. Study Population

Twenty-two patients diagnosed with POTS based on current consensus criteria [2,3] who were regularly engaged in work activity were consecutively enrolled by the Cardiovascular Autonomic Disorders Unit at Humanitas Research Hospital between August 2016 and December 2018.

Eighteen sex- and age-matched healthy subjects with a work activity similar to that of the patients participated in the study as controls to compare WAI scores in POTS patients and healthy individuals.

All POTS patients were studied during washout from drugs affecting cardiovascular autonomic control—i.e., betablockers, ivabradine and midodrine. They were asked to refrain from caffeine on the day of the study. Alterations in cardiac function of all POTS patients enrolled were excluded based on echocardiogram investigations.

The demographic and clinical features of POTS patients and controls are reported in Table 1.

### 2.2. Clinical Profile and Questionnaires

POTS patients and controls underwent a complete clinical evaluation. The POTS patients’ actual autonomic symptoms were assessed by administering the 31-item Composite Autonomic Symptom Score (COMPASS31) [28]. This questionnaire explores six domains of autonomic functions (orthostatic intolerance, vasomotor, secretomotor, gastrointestinal, bladder, and pupillomotor). Single domain scores are combined into a total score which ranges from 0 (normal) to 100 (the worst condition) [28].

Every POTS patient and control filled out the Work Ability Index (WAI) questionnaire [26]. This is a validated tool [29,30,31,32,33], which furnishes a self-assessment of employees’ work ability. The index is determined based on the answers to a series of questions that take into consideration the demands of work, the worker’s health status and resources. The physician in charge rated the responses according to seven items: 1. current work ability (WA) compared to the lifetime best (range 0–10); 2. WA in relation to the job’s demands (range 2–10); 3. current diseases diagnosed (range 1–7); 4. estimated work impairment (range 1–6); 5. sick leave in the last year due to diseases (range 1–5); 6. own prognosis of WA 2 years from now (score 1,4,7); 7. mental resources (range 1–4). The total WAI score range is 7–49. Ultimately, this tool assigns patients to 4 categories based on their ability to work: poor (7–27), moderate (28–36), good (37–43), and excellent (44–49). The WAI also provides a quantitative evaluation of comorbidities.

### 2.3. Cardiovascular Autonomic Profile of POTS Patients

The cardiovascular autonomic profile of POTS patients was assessed while supine and during a 75° head-up tilt (HUT) in the presence of stable hemodynamic parameters (heart rate and arterial pressure) and in the absence of pre-syncope symptoms and signs (nausea, sweating, and pallor).

An electrocardiogram (Dual Bio Amp, AD Instruments PtY Ltd., Bella Vista, Australia) and non-invasive beat by beat arterial pressure (Nexfine, SEDA SPA, Trezzano S-Naviglio, Italy) were continuously recorded for 20 min in the supine position and during a 15-min HUT. Respiratory activity was concomitantly evaluated by the thoracic bellows connected to a pressure transducer (Respiratory Belt, FM, Marazza, Italy). A catheter was placed on the non-dominant arm for blood withdrawal.

After the steps described above, each subject was placed on a motorized tilt table. Twenty minutes after adaptation to the experimental setting, baseline data acquisition was initiated and a first venous blood sample was collected to assess plasma catecholamines. Thereafter, the subjects underwent a 75° head-up tilt for 15 min. After 5 min of the tilt procedure, a second venous blood sample was taken. Plasma norepinephrine and epinephrine were determined by high-performance liquid chromatography with electrochemical detection [34].

### 2.4. Data Analysis

Data analysis was performed while supine after twenty minutes of baseline and after 5 min of HUT. The time series length was fixed at 300 consecutive beats in both conditions. This length was identified as the best compromise between the need for a large time series, in order to achieve greater accuracy in the computation, and the need to obtain stationary recordings, which would be easier for short time periods [35,36].

A power spectrum analysis of RR intervals, arterial pressure and respiratory activity variabilities was performed to obtain the indexes of cardiovascular autonomic profiles of POTS patients while supine and during HUT.

The heart period was approximated as the temporal distance between two successive R-wave peaks detected on the electrocardiogram. Electrocardiogram, arterial pressure and respiratory activity were digitized at 400 Hz by an analog-to-digital converter and recorded by a data acquisition system (PowerLab 16/35 and LabChart-pro 8.0 software; ADInstruments Pty Ltd., Bella Vista, Australia) for off-line analyses.

Principles of autoregressive spectrum and cross-spectrum analyses of RR interval, systolic arterial pressure (SAP) and respiratory activity variabilities have been described in detail elsewhere [35,36,37,38].

Briefly, from the electrocardiogram signal the continuous series of RR intervals (tachogram) were obtained by a derivative/threshold algorithm using the peak value of the R wave as a fiducial point. The selected sequence over the original series was tested for stationarity after linear detrending [39]. A new sequence of RR intervals was selected, in cases where the test for the steadiness of mean and variance were not fulfilled, until the prerequisites for restricted weak stationarity were obtained [39]. The Levinson-Durbin recursion provided the autoregressive model coefficients and the variance of the white noise. Akaike’s figure of merit automatically furnished the number of coefficients ranging between 8 and 14 [35].

Two major oscillatory components can be identified by the spectrum analysis of the RR intervals, which showed spontaneous variability [35,36]. The first one is the high-frequency component (HF_RR_ 0.15–0.50 Hz at rest). This is synchronous with the respiratory activity and is a recognized index of the vagal modulation to the sinoatrial node discharge [35,36]. The second spectral component is the low-frequency one (LF_RR_, 0.04–0.15 Hz). The latter, when expressed in normalized units (n.u.), is an index of the sympathetic efferent modulation to the sinoatrial node and its changes [35,36,37].

The two oscillatory components of RR variability are presented both in absolute units (ms^2^) and normalized units (n.u.). The absolute values of each component correspond to the integral of the oscillatory components—LF_RR_ and HF_RR_. The normalized units of the oscillatory components were obtained by dividing the absolute power of each component by the total variance minus the power of the very-low frequency component (<0.03 Hz) and then multiplying by 100 [35,40,41]. The LF/HF ratio, a non-dimensional index, was also calculated to assess the reciprocal changes of sympathetic and vagal modulation of the sinoatrial node discharge [35,38,42,43].

Systolic arterial pressure (SAP) was computed as the maximum of the arterial pressure in a given heart period [44]. The low frequency (LF) oscillatory component of SAP variability (LF_SAP_, ≈0.1 Hz) is taken as a marker of the sympathetic modulation of vasomotor activity [35,38,45,46] and is expressed in mmHg^2^. Spectral analysis has also been performed on the respiratory signal to assess the main respiratory frequency [35].

The cardiac baroreflex sensitivity was assessed by a frequency domain approach at rest and during HUT by the alpha index (α) [35,47,48] as the square root of the ratio of the LF_RR_ to LF_SAP_ and expressed in ms/mmHg.

### 2.5. Statistics

Continuous variables are expressed as mean ± standard error (SE), while categorical variables are expressed as numbers and percentages. The normality of the data was tested via the Shapiro-Wilk test. A Student’s unpaired-samples *t*-test was used to compare POTS patients and controls. The Student’s paired-samples *t*-test was used to compare baseline and HUT in POTS patients.

A Spearman correlation analysis was performed to determine the relationships between WAI and COMPASS31 total scores, and between WAI scores and individual changes of cardiovascular autonomic parameters induced by HUT in POTS patients. The correlation coefficient r, the type I error probability *p* and the confidence interval (CI) were calculated. The level of significance was set at 0.05. Statistical analyses were conducted using GraphPad PrismTM Software, version 8.00 (GraphPad Software, San Diego, CA, USA).

## 3. Results

Both POTS patients and controls were regularly engaged in work activities at enrollment.

### 3.1. Clinical Features and Work Ability in POTS and Controls

As reported in Table 1 and Table 2, the working capacity as assessed by the WAI was significantly reduced in POTS patients compared to controls. Of interest, all of the seven items explored by the questionnaire indicated a higher working impairment in POTS patients compared to controls (Table 2). The POTS comorbidities were mostly related to musculoskeletal (77%, back and cervical pain with or without degenerative disc disease and arthrosis), gastrointestinal (60%, functional gastrointestinal and irritable bowel diseases) and nervous system (50% headache, anxiety-depression and sleep abnormalities) disorders. Hypermobile Ehlers-Danlos syndrome and fibromyalgia were present in 23% and 18% of POTS patients, respectively (Table 1). Mitral prolapse was reported by four POTS patients.

During daily life, 91% of POTS patients were receiving pharmacological therapy (Table 1), and in most of the cases the drugs aimed to reduce HR (betablockers and ivabradine, 27%). Anxiolytics and analgesics were regularly used by 23% and 27% of patients, respectively. Work activity in 68% of POTS patients was characterized by both mental and physical demands and by a prevailing physical demand in 6% of POTS patients.

**Figure 1:**
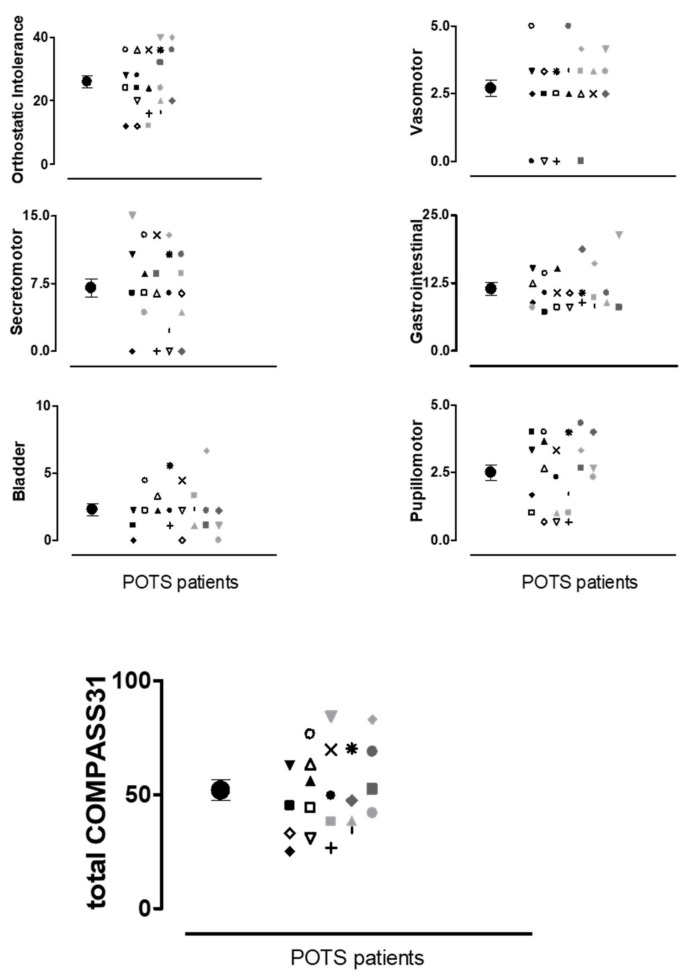
Individual values of autonomic symptoms intensity for the six domains explored by the Composite Autonomic Symptom Score (COMPASS31) questionnaire and the total COMPASS31 score. Each symbol in the graphs (i.e., *, x, Δ, …) represents the single POTS patient. The full black circles indicate the mean values of the scores with standard error bars.

The burden of autonomic symptoms assessed by the COMPASS31 in POTS patients is shown in Figure 1. The individual values for each domain explored and the total COMPASS31 score (bottom panel) are reported together with the mean values and error bars. The data indicate that the POTS patients are characterized by a wide range of autonomic symptoms of moderate-severe intensity.

### 3.2. Cardiovascular Autonomic Profile in POTS Patients

The indexes of cardiac and vascular autonomic control were obtained in 19 out of 22 POTS patients while supine and during 75° HUT and are reported in Table 3. In keeping with the diagnostic criteria, POTS patients were characterized by cardiac sympathetic predominance at baseline, as assessed by the LF n.u. and LF/HF indices. During HUT, a significant increase in the HR, LF/HF, and LF_SAP_ index of vascular sympathetic modulation and of plasma norepinephrine were observed. The mean SAP values remained unchanged. The mean increase in HR and LF/HF induced by HUT was 32 ± 2 from 75 ± 3 beats/min and 8.3 ± 2.6 from 3.5 ± 0.8, respectively.

The mean respiratory rate was in the normal range while supine, since it was similar to that observed in previous studies by our group [23,49], and remained unchanged during HUT. All POTS patients concluded the 15-min HUT without symptoms and signs of pre-syncope or syncope, in the presence of stable hemodynamic parameters.

### 3.3. Autonomic Impairment of POTS Patients and Its Impact on Work Ability

**Figure 2:**
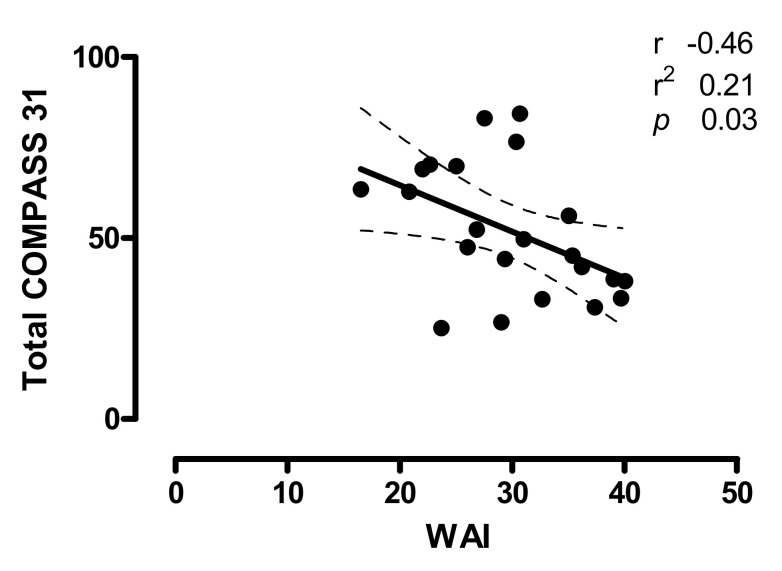
Relationships between the individual total COMPASS31 scores and the Work Ability Index (WAI) in POTS patients; r indicates the correlation coefficient, r^2^ indicates the goodness of fit and the dashed lines represent the 95% of confidence intervals. *p* < 0.05 is considered statistically significant. Notice that the higher the Total COMPASS31 score the lower the WAI score.

The relationships between the burden of autonomic symptoms of POTS patients, as quantified by the individual total COMPASS31 score, and the WAI are shown in Figure 2. Notice that a significant inverse correlation was present between the total COMPASS31 and WAI scores (r = −0.46; CI −0.74 to −0.04; *p =* 0.03). Indeed, the higher the total COMPASS31 score the lower the WAI score.

**Figure 3:**
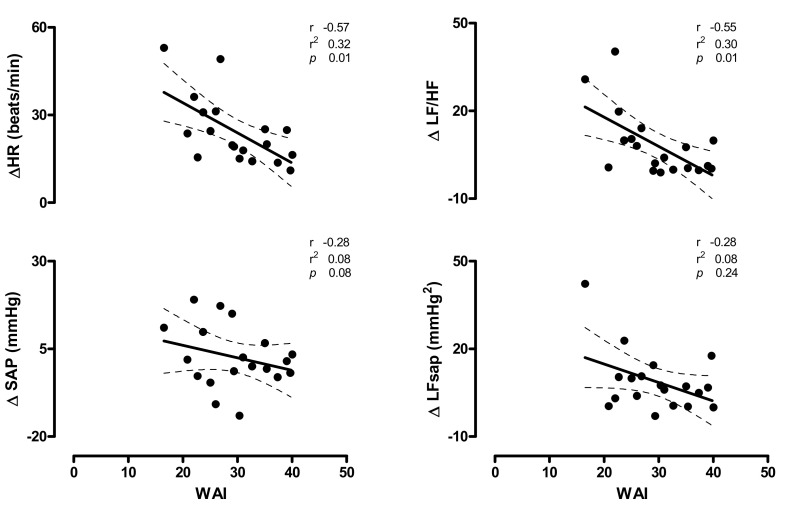
Relationships between the individual changes induced by 75° head-up tilt of heart rate (HR), the marker of cardiac sympatho-vagal modulation (LF/HF) (upper panels), systolic arterial pressure (SAP) and the marker of vascular sympathetic modulation (LF_SAP_) (bottom panels) and the Work Ability Index (WAI) in POTS patients; r indicates the correlation coefficient, r^2^ indicates the goodness of fit and the dashed lines represent the 95% of confidence intervals. *p* < 0.05 is considered statistically significant. Notice that the higher the increase in HR (∆HR, upper left panel) and LF/HF (∆LF/HF, upper right panel), the lower the WAI score.

**Figure 4:**
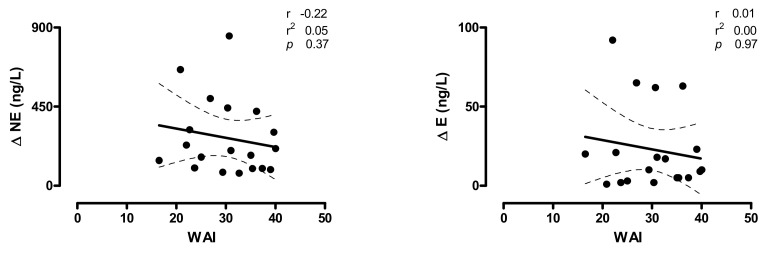
Relationships between the individual changes induced by 75° head-up tilt on Norepinephrine (NE) and Epinephrine (E) plasma values and the Work Ability Index (WAI) score in POTS patients; r indicates the correlation coefficient, r2 indicates the goodness of fit, the dashed lines represent the 95% of confidence intervals. Notice that a mild, non-significant relationship is present between the increase in NE induced by the HUT (∆NE) and the WAI score, while the increase in epinephrine induced by tilt (∆E) results unrelated with the WAI score of the POTS patients.

In Figure 3 (upper panels), the relationships between individual values of HR and the markers of cardiac sympatho-vagal modulation changes induced by HUT (∆HR and ∆LF/HF) and WAI score are depicted. A significant inverse relation was found between individual WAI scores and HUT increase in HR (r = −0.57; CI: −0.82 to −0.14; *p* = 0.01) and LF/HF (r = −0.55; CI: −0.81 to −0.12; *p* =0.01). A mild, non-significant, inverse relationship was observed between WAI- and the HUT-induced changes of the SAP (∆SAP), the marker of sympathetic vasomotor control (∆LF_SAP_) (Figure 3, bottom panels) and norepinephrine values (∆NE) (Figure 4, left panel).

## 4. Discussion

The main results of the present study indicate that our POTS patients, although regularly engaged in work activity, are characterized by a moderate-severe burden of autonomic symptom intensity, and by a concomitant reduced global work ability compared to healthy controls. In addition, the severity of the autonomic impairment, as assessed by the individual total COMPASS31 score, and the cardiac sympathetic activation induced by the orthostatic stimulus were inversely correlated with the Work Ability Index score in POTS. This suggests that, in our group of POTS patients, the greater the global autonomic impairment and the cardiac sympathetic overactivity during orthostasis, the lower the capability to work.

### 4.1. Work Ability and POTS

To the best of our knowledge, the present study is the first to explore the work ability in a selected group of adult patients affected by POTS who actively work by using a validated tool—i.e., the WAI questionnaire [29].

As recently underlined by Ilmarinen [29], the reduction in work ability is a phenomenon that involves both work organization and management aspects and the decline of human resources in a complex relationship model [29]. For these reasons generic interventions aimed at improving working ability in the workplace have been, until now, less effective than expected [50].

Previous research reported that POTS patients suffer from significant and debilitating symptoms that significantly affect their quality of life [14,15,16,51] in the presence of disability and associated comorbidities. As a consequence, about one-third of POTS patients become unable to continue their studies or maintain their job activities [14,15,16,22,51]. However, evidence on how POTS may influence work capability is lacking [2,3,16].

The work ability of the POTS patients in the present study was significantly impaired compared to age- and sex-matched controls. This was seen for work activities characterized by both physical engagements, including prolonged standing, and mental tasks. Indeed, the symptoms reported by POTS patients, particularly during the up-right posture, are characterized by palpitation, fatigue, dizziness, pre-syncope and, rarely, syncope [2,5] which probably affect most physical demands. Light-headedness, vision disturbance and concentration difficulty may negatively influence work tasks which require greater mental load as it may occur in white-collar workers. In addition, the work environment might expose the patients to additional factors exacerbating the orthostatic intolerance symptoms—i.e., excessive heat exposure, intensive physical exertion and stressful conditions [2,3], thus producing further working disability.

In keeping with previous studies results [3,6,9,15,30,52,53], our POTS patients were also characterized by several comorbidities besides the disorders affecting the cardiovascular system. Indeed, all POTS patients reported to have at least one such comorbidity, while this was reported in only 39% of controls (Table 1). Among these, musculoskeletal disorders, fibromyalgia, migraines, sleep disorders and anxiety were the most common. Not surprisingly, this complex pathway may concur in terms of producing frailty and lead to early leave of employment, if not adequately managed.

The use of the WAI enabled us to furnish some new additional information about the potential effects of this chronic syndrome on the complex phenomenon that is the work performance [29].

Indeed, the WAI is a validated tool widely applied in the occupational setting including research studies. It is particularly used to assess the progressive reduction in working ability produced by ageing or by various causes of disability [24,25,26,29,32,54].

### 4.2. Burden of Global Autonomic Impaiment and Work Ability in POTS

The burden of autonomic symptoms in POTS was previously described in cross-sectional studies [14,16,52,55]. A recent study from Dipaola and colleagues [56] evaluated the intensity of different autonomic symptoms of a group of POTS patients at baseline and during a 2-year follow-up by using the COMPASS31 score. Data indicated an improvement of the overall score after two years along with a reduction in quality of life impairment. However, the difficulties reported by the patients concerning the work place did not change. The intensity of autonomic symptoms as assessed by COMPASS31 may be used as an index of the disease severity as recently reported by some authors [28,52,56].

An important observation of the present study is the inverse relationship between the burden of autonomic asymptoms as quantified by the COMPASS31 score and the WAI. Indeed, the higher the autonomic symptom intensity, the lower the WAI score. In keeping with previous studies, the most frequently reported symptoms by our POTS patients were those related to orthostatic intolerance, as expected, followed by gastrointestinal, secretomotor and pupillomotor symptoms. These data indicate a large involvement of the autonomic nervous system of moderate-severe intensity in our patients (Figure 1) and that this has as negative impact on their global work ability.

### 4.3. Cardiovascular Autonomic Response to Orthostatic Stimulus and Work Ability in POTS

In the present study, POTS patients were exposed to an orthostatic stimulus was and their cardiovascular autonomic profile was assessed. The up-right body position represents one of the most relevant physiological stimuli in humans and its maintenance over time is fundamental to guarantee an adequate active daily life. Many work activities require the standing up position or the frequent change from sitting to standing. Of interest, in POTS patients, the symptoms of orthostatic intolerance—i.e., excessive orthostatic tachycardia associated with intolerable palpitations, lightheadedness, weakness, blurred vision, nausea, tremulousness, anxiety and pre-syncope—are very common [1,2,3]. Even though the pathophysiologic classification of POTS encompasses different phenotypes, including the neuropathic type [8], a prevalent cardiovascular sympathetic tone while supine and a further excessive increase during standing is common in these patients [4,23]. Furthermore, in the presence of an unfavorable working environment—i.e., excessive heat exposure, stressful conditions or excessive physical demand—the working activity may become unsustainable and the risk for these patients to leave their job and to experience progressive physical deconditioning is real [57,58].

According to these considerations, our POTS patients, who are still regularly engaged in working activities, showed a predominant cardiovascular sympathetic tone while supine (Table 1) in the presence of normal baroreflex sensitivity and a marked increase in the indexes of cardiac sympathetic modulation when exposed to a gravitational stimulus—i.e., during head-up tilt. This may represent the most important cause of the orthostatic intolerance reported by the POTS patients.

Similarly to what has been observed for the burden of autonomic symptoms, the work ability as assessed by the WAI score was inversely correlated with the increase in HR and of the marker of cardiac sympatho-vagal balance (LF/HF) produced by the orthostatic stimulus (Figure 3, upper panels). Indeed, the higher the increase oinf both HR and cardiac sympatho-vagal modulation index, LF/HF, the lower the self-reported work ability, as quantified by the WAI.

The increase in the indexes of vascular sympathetic modulation (LF_SAP_) and of plasma catecholamines induced by HUT show only a slight, not statistically significant, correlation with the individual WAI score (Figure 3, bottom panels). This is not surprising considering that the primary impaired organ in POTS, responsible for most of the orthostatic intolerance symptoms, is the heart and its autonomic neural control [1,2,3,6,7,23]. In addition the increase in plasma cathecolamines during tilt in POTS patients is characterized by a different pathway according to the prevailing pathophysiological mechanism underlying the syndrome [3,4,6,8,10,11,13]. We believe that this might be the reason why no significant correlation were documented with the WAI.

An interesting issue of the present study is that the WAI seems to be strictly correlated with the intensity of the cardiac sympathetic overactivity in POTS. Finally, the treatment of POTS [3], which is aimed at reducing the sympathetic overactivity, might result in an improvement in work performance. The combined use of physical re-conditioning programs [10,58,59], the increase in water and salt intake [2,3], the administration of ivabradine [60,61] or low dosage of betablockers [2,3] may help in accomplishing such an task. The atrioventricular node ablation and pacemaker implantation should be limited to highly selected POTS patients with severe recurrent syncope [62].

All of the above aspects become crucial in order to prevent early retirement and long-term sickness leaves, particularly in people with chronic physical disabilities [27,63].

## 5. Study Limitations

In the present study, the cardiovascular autonomic profile has been assessed only in POTS but not in controls. However, the present study mainly focused on the POTS patients, and in particular on the relationships between the intensity of disease and work ability.

Additionally, the small size of the study population and the different clinical phenotypes and disease duration of POTS patients may have partially affected the results; thus, our findings need further corroboration.

Finally, it is well known that anxiety and psychological distress are common in POTS. This could have produced an overestimation of some self-reported symptoms.

## 6. Conclusions

The total WAI score showed a reduced working ability in POTS patients and identified comorbidities that may promote frailty at work.

In this group of active workers with POTS, the WAI score was inversely correlated with both the intensity of the autonomic disease symptoms and the level of cardiac sympathetic activation induced by the gravitational stimulus. Indeed, the higher the burden of autonomic symptoms, the lower the WAI. In addition, the cardiac sympathetic overactivity during standing seems to play a negative role on work capability and performance.

Finally, the assessment of the characteristics of work impairment of POTS patients by the different WAI items might facilitate the implementation of more focused interventions in the workplace to adapt work demand and environment to the individual patient’s clinical profile. This should facilitate the working activity maintenance of patients affected by POTS, reduce their physical deconditioning [57,58] and promote a better prognosis.

A holistic approach involving a specific autonomic evaluation, an assessment of the cardiovascular autonomic profile and the work ability of patients suffering from POTS, might promote therapeutic and preventive interventions also in the workplace aiming to keep these patients active, reduce leave of employment and improve the long-term prognosis.

## Figures and Tables

**Table 1:** Demographics and Clinical Characteristics of Postural Orthostatic Tachycardia Syndrome (POTS) patients and Controls.

Demographics and Clinical Characteristics	POTS Patients (*n* = 22)	Controls (*n* = 18)
Age (years)	36 ± 2	39 ± 3
Sex (Male/Female)	2/20	4/14
Body Mass Index (kg/m^2^)	21.2 ± 0.7	21.9 ± 1.0
Total WAI score	29.8 ± 1.4	45.6 ± 0.5 *
Coexisting diseases, *n* (%)	22 (100)	7 (39) *
Cardiovascular	7 (32)	-
Musculoskeletal	17 (77)	2 (11)
Gastrointestinal	13 (59)	-
Nervous System	11 (50)	-
Ehlers-Danlos syndrome	5 (23)	-
Fibromyalgia	3 (14)	-
Other	19 (86)	6 (27)
Drug assumption, *n* (%)	20 (91)	2 (11) *

WAI indicates the Work Ability Index. Results are expressed as mean ± standard error. * *p* < 0.01 POTS vs. controls. Note that the subset of participants had several diseases, and total numbers of participants when adding for each disease does not match with the total numbers of coexisting diseases.

**Table 2:** The seven items explored by the Work Ability Index (WAI) and the total WAI score in POTS patients and controls.

Work Ability Domains	POTS Patients (*n* = 22)	Controls (*n* = 18)	*p* Value
Current work ability compared to the lifetime best (0–10)	5.68 ± 0.39	9.06 ± 0.22	*p* < 0.01
Work ability in relation to the job demands (2–10)	6.73 ± 0.32	9.44 ± 0.22	*p* < 0.01
Current diseases diagnosed (1–7)	2.68 ± 0.31	6.50 ± 0.17	*p* < 0.01
Estimated work impairment (1–6)	3.50 ± 0.22	5.89 ± 0.08	*p* < 0.01
Sick leave in the last year due to the diseases (1–5)	2.98 ± 0.24	4.28 ± 0.11	*p* < 0.01
Own prognosis of work ability two years from now (1,4,7)	5.91 ± 0.37	7.00 ± 0.00	*p* = 0.01
Mental resources (1–4)	2.36 ± 0.18	3.46 ± 0.10	*p* < 0.01
Total WAI score	29.84 ± 1.40	45.63 ± 0.53	*p* < 0.01

Results are expressed as mean ± standard error.

**Table 3:** Indices of cardiac and vascular autonomic profile of POTS patients (*n* = 19) while supine and during 75° head-up tilt (HUT).

Parameter	SUPINE	HUT
RR (ms)	806 ± 30	611 ± 20 *
HR (beats/min)	75 ± 3	107 ± 3 *
RRvar (ms^2^)	2582 ± 746	803 ± 139 *
LF_RR_ (ms^2^)	810.0 ± 227.7	337.4 ± 88.9 *
LF_RR_ (n.u.)	61.1 ± 5.5	78.2 ± 3.1 *
HF_RR_ (ms^2^)	961.8 ± 424.7	64.3 ± 22.0 *
HF_RR_ (n.u.)	36.2 ± 5.5	15.2 ± 2.9 *
LF/HF	3.5 ± 0.8	11.7 ± 2.8 *
SAP (mmHg)	108 ± 3	110 ± 3
DAP (mmHg)	66 ± 2	71 ± 2 *
LF_SAP_ (mmHg^2^)	3.2 ± 0.7	12.0 ± 2.6 *
RESP (cycles/min)	17.3 ± 0.5	16.9 ± 0.7
αLF (ms/mmHg)	21.3 ± 4.1	5.7 ± 0.9 *
NE (ng/L)	252.1 ± 32.3	522.0 ± 71.4 *
E (ng/L)	27.4 ± 3.7	51.3 ± 7.6 *

RR indicates R-R interval; HR, Heart Rate; RRvar, RR variance; LF_RR_, low frequency power of RR variability; HF_RR_, high frequency power of RR variability; n.u., normalized units; LF/HF, ratio of the LF power to the HF one of RR series; SAP, Systolic Arterial Pressure; DAP, Diastolic Arterial Pressure; LF_SAP_, low frequency power of SAP variability; RESP, respiration rate; αLF, index of arterial baroreceptor sensitivity assessed by bivariate power spectrum analysis; NE, plasmatic norepinephrine; E, plasmatic epinephrine. Results are expressed as mean ± standard error. * *p* < 0.05 SUPINE vs. HUT.
